# Recognising the differences in the nurse consultant role across context: a study protocol

**DOI:** 10.1186/1472-6955-13-30

**Published:** 2014-10-13

**Authors:** Michelle Giles, Vicki Parker, Rebecca Mitchell

**Affiliations:** 1 Hunter New England Local Health District, James Fletcher Campus, 72 Watt Street Newcastle, Newcastle, NSW 2300, Australia; 2 School of Health, University of New England, Armidale, NSW, Australia; 3 School of Business and Law, University of Newcastle, Callaghan, NSW 2308, Australia

**Keywords:** Nurse consultant, Nursing, Workforce, Role clarity, Advanced practice nurse

## Abstract

**Background:**

The advanced practice role of the Nurse Consultant is unique in its capacity to provide clinical leadership across a range of contexts. However, the Nurse Consultant role has been plagued with confusion due to lack of clarity over function and appropriateness for purpose within health organisations across contexts. Changing health service delivery models are driving the emergence of new nursing roles, further clouding the waters related to role positioning and purpose. There is an urgent need for evidence of impact and demonstration of how Nurse Consultants contribute to health care outcomes. This study aims to gain a clearer understanding of the Nurse Consultant role and its impact in metropolitan and rural New South Wales (NSW) Australia.

**Design:**

The proposed study employs a sequential mixed method design, underpinned by Realistic Evaluation, to explore how Nurse Consultants contribute to organisational outcomes. The ‘context – mechanism – outcome’ approach of realistic evaluation provides a sound framework to examine the complex, diverse and multifaceted nature of the Nurse Consultant’s role.

**Method:**

Participants will be stakeholders, recruited across a large Local Health District in NSW, comprising rural and metropolitan services. A modified and previously validated survey will be used providing information related to role characteristics, patterns and differences across health context. Focus groups with Nurse Consultant’s explore issues highlighted in the survey data. Focus groups with other clinicians, policy makers and managers will help to achieve understanding of how the role is viewed and enacted across a range of groups and contexts.

**Discussion:**

Lack of role clarity is highlighted extensively in international and Australian studies examining the role of the Nurse Consultant. Previous studies failed to adequately examine the role in the context of integrated and complex health services or to examine the role in detail. Such examination is critical in order to understand the significance of the role and to ascertain how Nurse Consultants can be most effective as members of the health care team. This is the first Australian study to include extensive stakeholder perspectives in order to understand the relational and integrated nature and impact of the role across metropolitan and rural context.

## Background

Health care is currently facing enormous challenges across the globe with ageing populations, increased chronic diseases and workforce shortages in an environment of massive reforms and scarce health dollars [1-3]. The number of Advanced Practice Nurses (APN) is increasing internationally in response to these changes, creating different roles with an emphasis on service replacement, gap filling and expanded scope of practice [4]. There is however inconsistency in nomenclature for APN roles across countries [5,6]. This has certainly blurred the boundaries of role function leading to calls for role clarification as APN roles respond to current health service needs in what has been described as an adhoc manner [5,7-9].

There is an urgent need for examination of how the APN role of Nurse Consultant can be deployed, not necessarily in traditional ways, but in the most appropriate and effective manner that fits best with health care redesign that aims to improve patient outcomes through impact and efficiency in a way that is economically sustainable. To achieve this it is important that decision makers and health care team members have a clear understanding of each role within the health care team.

Internationally there has been extensive work done on determining the key attributes of a Nurse Consultant role and despite the many titles applied across the globe and inconsistent role application [10] many of these roles are similar in scope of practice and function [5,10,11]. The Nurse Consultant role in Australia aligns with international descriptions of the APN, in that it comprises several key attributes: clinical expertise, leadership, autonomy and role development [12]. The Nurse Consultant is able to provide expanded and autonomous clinical practice, make complex decisions (ICN, 2012; Jolienemi, Duffield, 2009), provides leadership, education and support at a clinical level to advance the practice of others and leadership at a strategic level to implement change and innovation in practice [10-12].

In international literature the Nurse Consultant role differs to the Nurse Practitioner (NP) in that their expert knowledge base is more likely to be applied to the education and development of practice in others and they are more likely to be involved in leading practice change and research [10]. Nurse Consultant roles are described as multidimensional, diverse and complex in nature [13-16] with several sub roles; practitioner, educator, consultant, clinical leader and researcher [13,17]. Jokiniemi et al. [11] in a systematic review identified consistencies in non-NP APN roles across countries when examining the Nurse Consultant in the UK, the Clinical Nurse Specialist (CNS) in the US and the Australian Nurse Consultant role.

In Australia the Nurse Consultant role is described and appropriated through domains of practice identified for example in the NSW Health Clinical Nurse Consultant – Higher Grades – Public Hospitals Nurses’ State Award, these are; Clinical Service and Consultancy, Clinical Leadership, Research, Education and Clinical Services Planning and Management. These domains are closely aligned to the UK model [11,18,19] where the Nurse Consultant role was implemented in 2000 to achieve better health outcomes and is identified by four core functions; expert practice, leadership and consultancy, education, training and service development and research and evaluation [20].

The proliferation of new nursing roles and continued ambiguity about role functions and boundaries internationally [11] have led to concerns about lack of role clarity and the need for distinction between various roles [4-6,8,9,16,21-23]. This lack of clarity has been identified as one of the most important factors hindering the Nurse Consultant’s effectiveness in practice [7-9,16,24,25] the progression of APN roles internationally and the ability to articulate role value [12] Lack of clarity has also contributed to inconsistency in the way the role of Nurse Consultant has been implemented both within Australia and internationally [5,6,10,11]. Difficulties also arise due to existence of discrete roles for each of the domains (educator, manager, care coordinators for example) that make up the single role of the Nurse Consultant [24].

Historically, little attention has been paid to examination and evaluation of the long standing Nurse Consultant type role and the nature of its evolution with changing health care services, particularly across contexts [5,26,27]. The nature of the Nurse Consultant as a complex multidimensional leadership and practice support role with in many cases no direct relationship to patient and practice outcomes makes it difficult to attribute patient outcomes to the role [25,28-30] and compounds role ambiguity. Without evidence of value and ‘fit for purpose’ the role remains invisible [12], undervalued by managers [31] and at risk of misappropriation and erosion [25,32].

There are a number of international UK based studies that attempt to identify the characteristics of the Nurse Consultant, explore the effectiveness and impact of the role on health service delivery, the personal attributes and the organisational influences on the role [18,19,33,34]. These studies highlight the difficulties of attribution of impact on patient care to the Nurse Consultant role the importance of organisational support structures and networks. A systematic review examining the impact of Nurse Consultant roles in adult healthcare settings [35] supports this view and concludes that evidence to date is unclear. This is attributed to the fact that impact is multifaceted and hard to capture due to the diverse and complex nature of the role in that many work as part of multidisciplinary teams and influence the practice of others [35,36]. There is evidence highlighted of impact in service development and clinical leadership [19,31], but the role tends to have an indirect impact on patient outcomes which is a challenge to evaluation [12]. An Irish study evaluating the advanced nurse practitioner [37] found that these roles promote client satisfaction and better self-management as well as provide a more holistic approach to assessment leading to earlier diagnosis and interventions.

A systematic review based on US studies examining APN outcomes across an 18 year period to 2008 also recognized the difficulties in outcome attribution to the CNS role due to the complexity of the intervention, however they did find that the use of the role in acute care reduced length of stay and cost of care for hospitalized patients [38].

In Australia, very few studies are specific to the Nurse Consultant, often combining the Nurse Consultant and the NP roles [24]. In line with international findings the key recommendations from the Chiarella et al. [24] review included the need for; role clarification as well as career development and capacity building opportunities, consideration of their support infrastructure; and clarification of the research component of the Nurse Consultant role.

In general, Australian studies have been relatively small and focused predominantly in metropolitan based or acute care context, focusing on roles with direct patient care in narrow specialty areas and making no distinction between role grades [21,39,40]. Some of these studies have highlighted the practice domains outlined in the Award [17] as ambiguous and verbose and not easily translated into practice [21,40]. The existing inconsistencies in actualization of the role have led to underutilization of the clinical leadership capacity of the Nurse Consultant as well as lack of support systems for them [39] have been limited to examination of the role in isolation and not as part of an integrated care service. A further limitation of previous studies is that here has been little stakeholder engagement to gain insight and understanding of the role as part of a health service team, nor how the role is viewed or valued by other professions. The positioning of the role in relation to current changing health service models and the current or potential function of the role in this context has not been considered in any previous Australian studies. In particular, there has been no differentiation between the nature of the role in metropolitan and rural context.

Clinical practice in a rural context differs markedly from that of metropolitan practice, geographically, sociologically and demographically [1,38,41,42]. Rural clinical practice requires greater diversity of skill and knowledge and is described as being more generalist in nature [43]. This is in an environment where clinicians can be sole practitioners working in isolation with minimal support structures [44] and lack of opportunity for professional development compounded by the need to travel long distances to deliver services to isolated communities [43]. To address the challenges facing healthcare professionals in rural acute care settings key strategy highlighted are being flexible in practice and expanding scope of practice to adjust to what is needed from the service [43]. Little is known about how the Nurse Consultant role function differs in metropolitan practice when compared to rural practice or when the role extends across both metropolitan and rural context.

It is critical that Nurse Consultants are clear about their scope, roles and functions and that this is articulated to other health professionals so that Nurse Consultants can be effective members of the health care team [4,7-9,16]. This can only be achieved with close examination of existing Nurse Consultant roles and strategic alignment with service and professional goals.

The research described in this protocol is designed to gain a clearer understanding of the role of Nurse Consultant across context, and in particular distinguishing between metropolitan and rural based Nurse Consultants. It will also focus more broadly on the impact of the role from a whole of service delivery perspective, incorporating the views and experiences of all key stakeholders including professionals across health disciplines, managers and Nurse Consultants. This will extend the knowledge gained from the limited amount of previous work done on the Nurse Consultant by identifying the unique nature of the role and how it is applied and enacted. This is the first Australian study to take a stakeholder approach in order to understand the relational and integrated nature and impact of the role within the different contexts of health service delivery and within health service teams.

### Aim of the study

This study aims to gain a clearer understanding of the Nurse Consultant role across contexts.

It will examine:

• Nurse Consultant role descriptions, policy guidelines and standards

• Nurse Consultant activity, scope of practice, patterns of role engagement, networks and relationships across health service delivery contexts in both metropolitan and rural areas.

• Stakeholder attitudes and views about the role and its’ contribution

• The match between actual and expected contribution.

## Design

**Figure 1:**
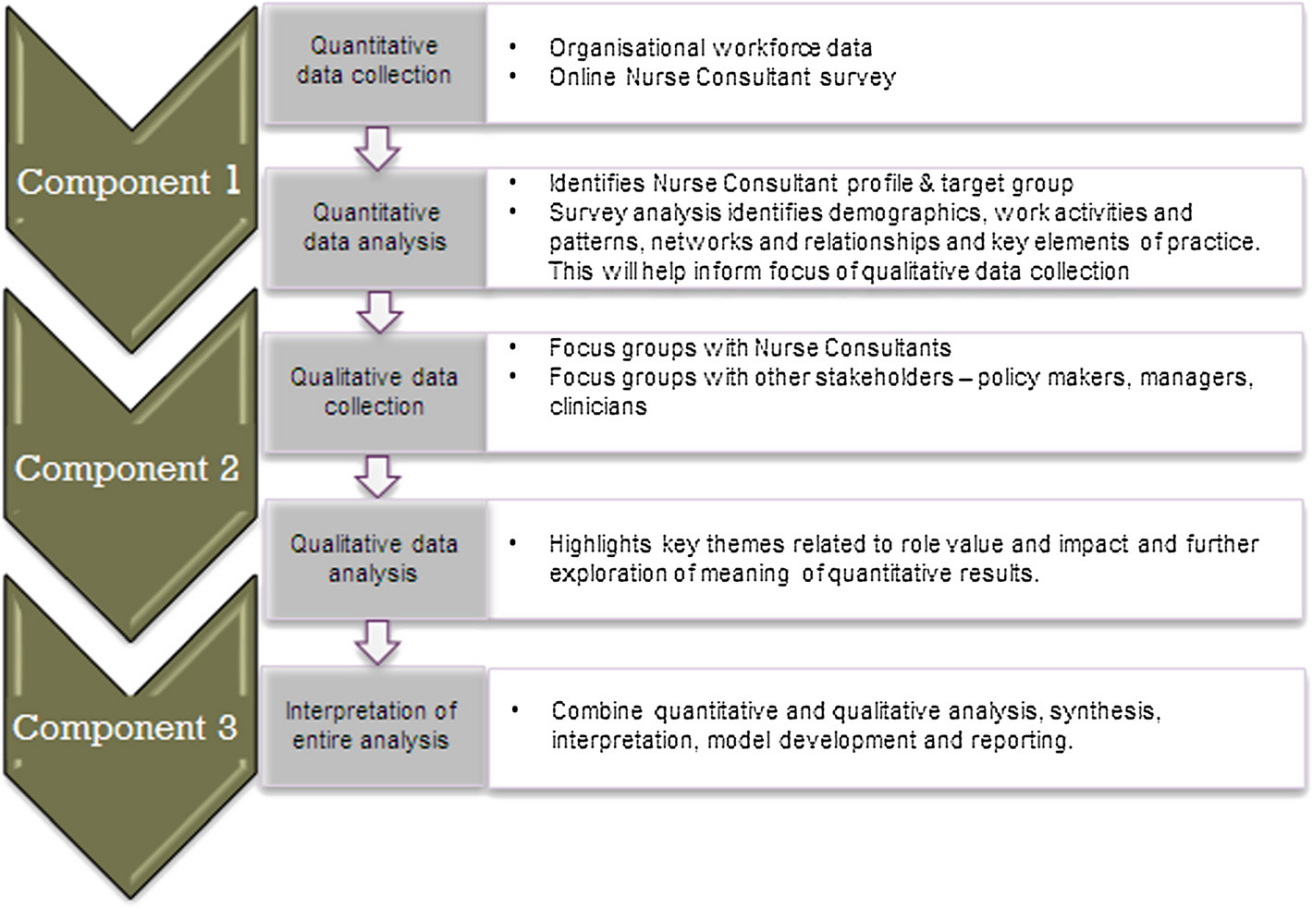
Components of the study.

This study uses a mixed method, cross sectional design with sequential and integrated quantitative and qualitative methods of data collection and analysis. Mixed method approaches provide a mechanism for understanding and explaining complex organisational and social phenomena [45] and provides for a more comprehensive and deeper understanding ([46-48]. In this study a mixed method sequential design provides for one phase to influence the focus of the next phase of the study and details are provided in Figure 1.

Mixed method research, in providing a richer and more complete view of reality, fits perfectly with examination of the complexities and diversities of the Nurse Consultant role and the past difficulties highlighted by researchers in examining all aspects of this multidimensional role [49].

### Research framework

The research framework of realistic evaluation underpins this study and employs an analytical approach to investigating causal mechanisms and how they work and under what circumstances [50]. Realistic evaluation has been highlighted as an effective framework for complex, multicomponent activities in health care [51].

**Figure 2:**
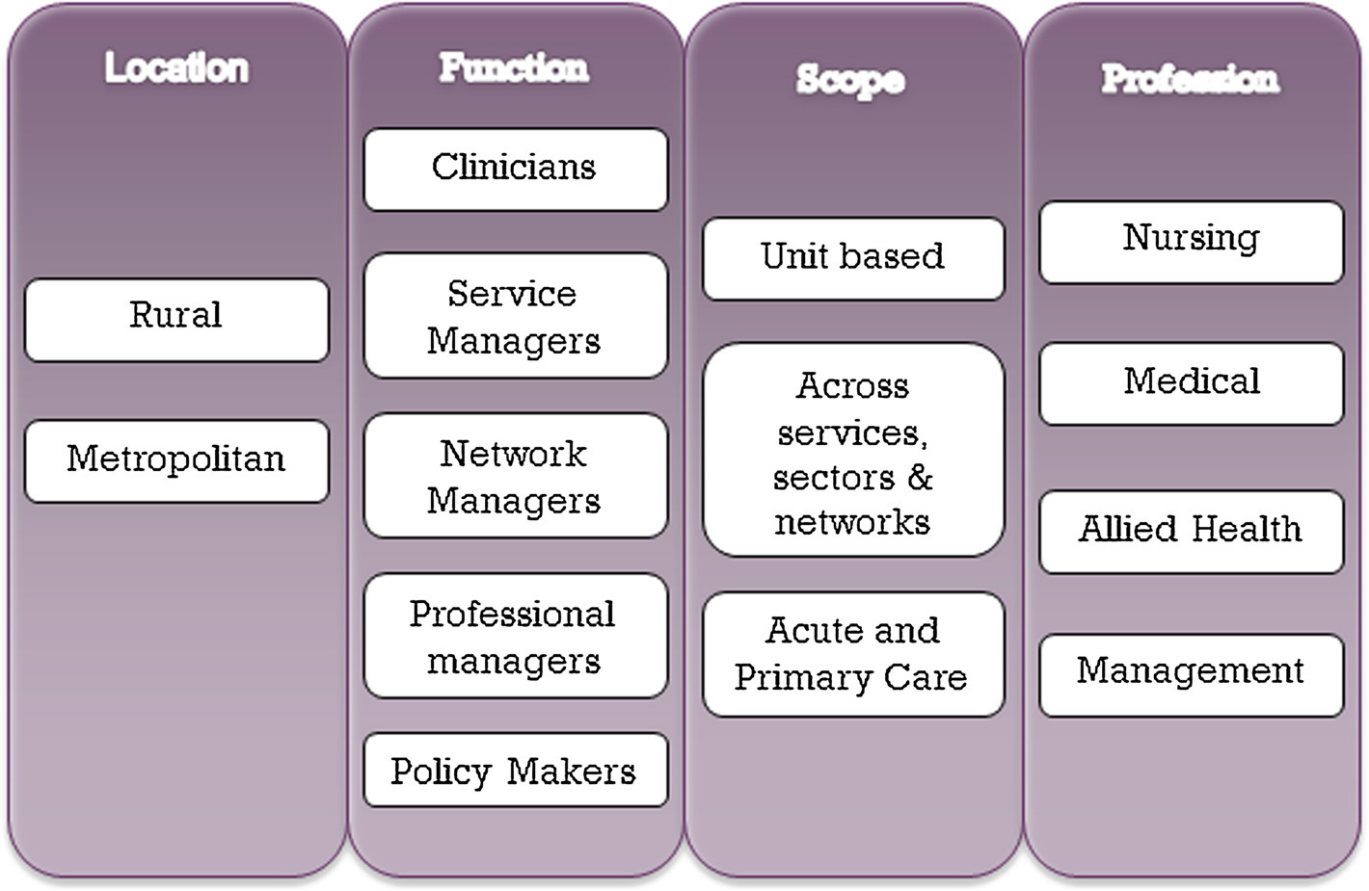
Stakeholder sampling strategy.

Realistic evaluation is underpinned by several broad principles [50];

• Stakeholder involvement and engagement,

• Mechanisms are contingent upon context.

• The development and testing of context, mechanism and outcome gives explanation about what works for whom, how and in what circumstances.These principles have guided the studies approach to stakeholder involvement as well as provided a framework for data analysis and comparison (Figure 2).

Because causal mechanisms are embedded within particular contexts and social processes, realistic evaluation acknowledges the need to “understand the complex relationship between these mechanisms and the effect that context has on their operationalization and outcome” [52]. This is summed up by Pawson et al. [53] as the CMO: Context - Mechanism – Outcome model, which incorporates contextual influences and mechanisms of effect. Realistic evaluation is concerned with exploring what mechanisms work, under what conditions, why and to produce what outcome [52].

Our research called for an understanding of how the Nurse Consultant role is being operationalized within the reality of different clinical context and what impact the role has on practice, practitioners, organisations and patients. This involves understanding the relationships between the types and nature of particular role applications (Mechanisms) in a variety of health service delivery settings (context) and what impacts this resulted in (Outcomes).

The generic principles and values of advanced nursing practice have been expressed by Bryant-Lukosius and Dicenso [54,55] as ‘coordinated care and collaborative relationships among health care providers and systems. A focus of this study is on gaining an understanding of the processes and mechanisms through which Nurse Consultants practice and function across both rural and metropolitan context, their networks and relationships and the supports or constraints that impact on the effectiveness of the role. This is well supported by much of the literature that examines the optimization of APN roles [5,16,24,26,33,56].

Based on this framework the survey of Nurse Consultant’s will provide understanding of role context, enactment, engagement, networks, role patterning, satisfaction levels and perceived role support. The sequential nature of data collection will allow analysis of survey data to inform focus groups with Nurse Consultant’s investigating in more detail perceptions of role value, barriers and facilitators to role effectiveness, relationships, role support and resource provision.

Our focus groups with policy-makers, managers and lead clinicians will provide understanding of how the Nurse Consultant is viewed and valued by other health professionals and what their expectations are of the role. These focus groups also aim to explore any tensions highlighted in the Nurse Consultant focus groups in terms of role application and identify and contextual, institutional and professional influences on role application and effectiveness in different health care delivery structures.

The CMO framework will inform data analysis [50]. Context will be explored in terms of rural versus metropolitan, health sectors, government and local policy and institutional environment, role clarity, current attitudes towards the role and individual approaches and accountabilities. Mechanisms will be investigated in terms of the processes, role characteristics, components, activities and patterns, supports and enablers, tensions and barriers. Outcomes will be explored at the level of organisation, management, team and clinician.

## Methods

An extensive literature review will be undertaken in the first instance to identify how best to examine the role and to assist in developing the constructs for data collection.This study has three distinct components emphasizing the sequential nature of data collection (Figure 1). Initially a workforce profile will be established from workforce databases and a comprehensive survey of the Nurse Consultant workforce will be completed. This data will be extensively analysed to identify key elements of practice which will be further explored in the focus groups with Nurse Consultants and other stakeholders.

### Sample and data collection

This study is set in a very large Local Health District (LHD) in NSW that has a combination of metropolitan, rural and remote services within its boundaries.

Workforce data will be explored to identify all employed Nurse Consultants demographics and where they are located across the district and in what specialty or service. Other APN roles that currently exist within the district will also be identified to provide a nursing profile.

All Nurse Consultants within the LHD will be targeted to participate in an online survey that will explore the actual nature and function of the role. Based on organisational workforce data identifying a total of 196 Nurse Consultants employed across the district the desired sample size for the survey with a power calculation that demonstrates a representative sample with a confidence level of 95% is 130. Interim analysis will determine representation across rural and metropolitan context. The survey tool was a modified version of that used by Guest et al. [18] in the UK which they used to evaluate the role of the Nurse Consultant and Health Visitor and consisted of 43 item with likert scale, and multiple choice responses. Sections included; role context, enactment and engagement covering the notion of role patterns that describes the level to which each of the domains is included in the role [18,21,40] satisfaction levels, role innovation, networks, collaboration and engagement [7,18,19]. Organisational factors such as; structures, social supports, bridges and barriers and resources that support the role [24,26] will also be explored as well as expectations and role clarity [33,35,57]. Role characteristics and behaviors included; perceived effectiveness and competency, job autonomy, clarity, stressors, conflict and career and growth opportunities. Four open ended questions invited comment on challenges and innovations within the role. Minor modifications were made to the survey in the activity and interaction sections. These were context specific and included the addition of some rural and clinical networks and stream based questions as well as some questions with an inter-professional practice focus.

All Nurse Consultant’s will also be invited to participate in one of four focus groups located in both metropolitan and rural locations and for those who cannot attend a de-identified feedback forum will be provided online so that Nurse Consultants can answer the focus group questions electronically. The focus group questions will further explore preliminary findings from the online survey where further in-depth information is needed to clarify findings. Focus group questions will also seek to better understand complexities and differences in context, more specifically rural versus metropolitan based roles and the Nurse Consultant’s perspective on how the role is supported and valued.

To encourage Nurse Consultant’s to participate in both parts of the data collection process a communication strategy will be established early and face-to-face information sessions will be given in order to address any concerns from potential participants.A purposive sample of other stakeholders will be sought for up to six focus groups and the stakeholder sampling strategy is outlined in Figure 2. This will consist of those who manage and run services, policy makers and senior clinicians across both rural and metropolitan based services, across professions and across a variety of context. This will ensure that the sampling strategy is adequate and will provide valuable insight into how the Nurse Consultant role is positioned and valued by others within their services as well as assist in identifying enablers and challenges.

### Data analysis

Quantitative analysis will include extensive descriptive summary to produce a demographic and geographic profile of the Nurse Consultant workforce and their roles. Partial least squares (PLS) structural equation modelling (SEM) will be used to investigate potential models, derived from constructs identified in the literature review, and assess their utility in predicting our dependent variable. PLS SEM has been shown to be robust to relatively small sample sizes and requires fewer cases than covariance-based SEM. Based on the predicted return rate and hypothesised model, we utilise the rule-of-thumb for PLS SEM sample sizes of at least equivalent to the larger of; a) ten times the number of the construct with the largest number of formative indicators in the outer model, or b) ten times the largest number of structural paths (arrows) that are directed to any variable in the inner model [58]. Partial least squares SEM, like Covariance Based SEM provides the capacity to assess both measurement and structural models. For the measurement model, PLS SEM generates factor loadings. Our measures use reflectively measured constructs, and we will assess whether indicators are all over .70 as they show that the construct explains more than 50% of the indicators variance. We will also investigate internal consistency reliability by ensuring that composite reliability is over 60 and below 95 [58].

To investigate individual hypotheses we will also use ordinary least squares regression analysis which will allow the generation of confidence intervals to assist in the interpretation of hypothesised direct and indirect effects and investigation of regions of significance for proposed moderators. Analysis will also include categorical analysis, factor analysis and bivariate and multivariate analysis where relevant. Comparisons will be made between metropolitan and rural based respondents.

In any particular focus group participants will either be all rural or all metropolitan based to allow isolation of data for analysis and comparison. Qualitative analysis will consist initially of content analysis where qualitative survey and interview data will be clustered, coded and categorized. This data will then be analysed using iterative thematic process to produce a description of shared and disparate experiences, issues and concerns.

### Ethical considerations

Humans Research Ethics approval has been granted at a District and State level through the Hunter New England Research Ethics and Governance Unit and the University of New England Research Ethics Committee. Survey respondents and focus group participants will be provided with details of the purpose prior to their participation, ensuring informed consent. They will also be assured that their privacy will be protected at all times and that their participation will not impact on their employment or treatment at work. Digital audio files of focus groups will be stored in a password-protected file and only be accessible to members of the research team. Assigning pseudonyms and using codes in reporting responses will maintain confidentiality.

### Rigour

The method utilised in identifying and deliberately selecting focus group participants in the stakeholder groups ensures we will gain access to important sources of knowledge related to particular aspects outlined in the objectives of this research [59]. The sequential approach to data collection design will identify key issues in the survey that will direct and refine the focus of the Nurse Consultant qualitative data collection component. As well the triangulated approach to data collection will provide evidence from a wide range of sources to strengthen the accuracy and reliability of information [59]. The use of a previously developed and validated survey [18] which incorporates all the constructs identified in the literature related to exploring the nature and function of the role will further strengthen data reliability.

The use of the CMO framework to guide the data analysis component of the study will provide a consistent approach to qualitative analysis and several members of the research team will individually use this framework to thematically analyse and compare their results. NVIVO software will also be used in the coding process with collaboration between research members.

## Discussion

Health care is facing enormous challenges in an environment of major health care reform, scares resources and ageing populations and workforces [1,2] and APN role numbers are increasing internationally in response to these changes. This is blurring the boundaries of role function as roles develop as service gap fillers in an adhoc manner [4] and recent literature continues to call for role clarification as APN roles [7-9,16]. For this reason it is imperative that the Nurse Consultant role functions be well understood by APNs themselves as well as other health professionals so these roles can be appropriately deployment ensuring they reach their full potential [4,7-9,16] and “best fit” within health care organisations. This can only be done through close examination of existing Nurse Consultant roles to provide clarity of role function which will enable roles to be strategically realigned, developed, supported and strengthened according to whether the role functions within a metropolitan or rural context or both.

The significance of this study can be considered from several perspectives. Firstly, this research looks directly at the Nurse Consultant role, not in isolation, but as part of the complex networks, relationships and teams that make up a variety of health service delivery models. Previous Australian studies have tended to combine the two APN roles in studies [24] ignoring other stakeholder groups and focusing on the Nurse Consultant role in isolation and not as part of an integrated health service team. Secondly, the study design includes a range of different stakeholder perspectives at various levels within the organisation. The deliberate selection of stakeholders such as policy makers, professional and service managers and other clinicians will provide access to an important source of knowledge not previously explored in relation to the Nurse Consultant. Thirdly, the use of a well-established model, the CMO model which incorporates a focus on process, support, enablers and barriers fits well with examination of complex and diverse components of the Nurse Consultant role and how it is integrated into services and teams. These approaches contribute to the rigour of our design and validity of analysis and findings and their ability to inform policy and practice initiatives. Lastly, data analysis based on structural pathway modelling will provide valuable information to assist in the development of frameworks and models for optimal practice. This form of data analysis will provide a truly unique insight into moderating and mediating factors for role effectiveness that have not been presented in any previous studies of this nature.

Given the majority of previous studies were set in a metropolitan context this study will pay particular attention to engaging rurally based stakeholders and identifying the differences in rural and metropolitan based roles in relation to role function and activities, patterns of engagement, networks and how the position is valued within the interprofessional teams. The barriers and enablers will also be examined to identify differences.

This study will extend knowledge by identifying the unique and diverse nature of the role and how it is enacted in a variety of contexts, in particular across metropolitan and rural service areas. Findings will also provide insight into the tensions and conflicts role incumbents may experience as a consequence of health reform and provide a model for optimal implementation and sustainability of the role.

The results of this study will inform the development and strengthening of advanced practice career pathways for nurses and the education that underpins them. It will provide guidance to workforce planners, service managers and health care teams, ensuring appropriate skill mix for optimal outcomes.

The development of a framework by which to articulate and analyze roles that will inform processes for role application, implementation and evaluation as well as how the role is best integrated and supported in context. It will also identify essential attributes for future succession planning to plan capacity building strategies to strengthen the role and its contribution to health care and patient outcomes.

### Limitations of the study

While the study findings will be derived from situations unique to Australia, evidence within the literature suggests similarities across countries [5,10,11]. A notable point is that this study is focused on one LHD with only the one overarching management and organizational structure, and although large with metropolitan, rural and remote health care context there is potential to sacrifice identifying some broader contextual findings across LHD’s.

## Competing interests

All authors declare that they have no competing interests.

## Authors’ contributions

MG contributed to developing research conception, design and methods as well as initial drafting of this manuscript. VP contributed to development of research design and methods and has significantly critically revised and contributed to content in this manuscript. RM contributed to developing design and methods and significantly critically revised and contributed to content in this manuscript. All authors read and approved the final manuscript.

## Authors’ information

Co authors: Vicki Parker and Rebecca Mitchell.

## Pre-publication history

The pre-publication history for this paper can be accessed here:

http://www.biomedcentral.com/1472-6955/13/30/prepub
